# Controlling the Morphology of Barrel-Shaped Nanostructures Grown via CuZn Electro-Oxidation

**DOI:** 10.3390/ma15113961

**Published:** 2022-06-02

**Authors:** Damian Giziński, Kristina Mojsilović, Anna Brudzisz, Urša Tiringer, Rastko Vasilić, Peyman Taheri, Wojciech J. Stępniowski

**Affiliations:** 1 Faculty of Advanced Technologies and Chemistry, Institute of Materials Science and Engineering, Military University of Technology, Kaliskiego Str. 2, 00908 Warsaw, Poland; anna.brudzisz@wat.edu.pl; 2 Faculty of Physics, University of Belgrade, Studentski Trg 12–16, 11000 Belgrade, Serbia; kristina.mojsilovic@ff.bg.ac.rs (K.M.); rastko.vasilic@ff.bg.ac.rs (R.V.); 3 Department Material Science and Engineering, Faculty of Mechanical, Maritime and Materials Engineering, Delft University of Technology, 2 Mekelweg, 2628 CD Delft, The Netherlands; u.tiringer-1@tudelft.nl (U.T.); p.taheri@tudelft.nl (P.T.)

**Keywords:** brass passivation, electro-oxidation, ZnO-CuO_x_ nanostructures, organic dye photodegradation

## Abstract

Herein, we report a feasible method for forming barrel-like hybrid Cu(OH)_2_-ZnO structures on α-brass substrate via low-potential electro-oxidation in 1 M NaOH solution. The presented study was conducted to investigate the electrochemical behavior of CuZn in a passive range (−0.2 V–0.5 V) and its morphological changes that occur under these conditions. As found, morphology and phase composition of the grown layer strongly depend on the applied potential, and those material characteristics can be tuned by varying the operating conditions. To the best of our knowledge, the yielded morphology of barrel-like structure has not been previously observed for brass anodizing. Additionally, photoactivity under both UV and daylight irradiation-induced degradation of organic dye (methyl orange) using Cu(OH)_2_-ZnO composite was explored. Obtained results proved photocatalytic activity of the material that led to degradation of 43% and 36% of the compound in UV and visible light, respectively. The role of Cu(OH)_2_ in improving ZnO photoactivity was recognized and discussed. As implied by both the undertaken research and the literature on the subject, cupric hydroxide can act as a trap for photoexcited electrons, and thus contributes to stabilizing electron-hole recombination. This resulted in improved light-absorbing properties of the photoactive component, ZnO.

## 1. Introduction

Brass-based composites have wide applications in various fields of modern material science. As a zinc-containing alloy a brass-based composite can be used as a substrate for preparation of semiconducting materials for photoinduced catalysis. When oxidized, it forms ZnO, which is a *p*-type semiconductor with wide band gap of 3.37 eV [1]. ZnO alone indicates absorption of light mainly from the UV region (only ~5% of visible light). Moreover, its ability to generate reactive agents during the photocatalytic process is limited due to the high rate of photo-generated charge carrier recombination [2,3,4]. Those drawbacks reduce the applicability of ZnO in solar energy-harvesting devices and motivate further exploration of modified Zn-containing bimetallic systems.

Copper- and zinc-derived oxide materials are used in many catalytic processes due to their accessibility, low price and versatility. It has been reported that CuZn-based systems efficiently catalyze CO_2_ reduction [5,6,7,8], organic pollutants degradation [9,10], and are used in electrochemical sensing [11]. Subjecting copper–zinc materials to oxidation yields very diverse oxide structures in terms of both chemical composition and morphological features. Combining ZnO with Cu oxidation products leads to formation of a cooperative material with tuned light-absorbing properties. For instance, CuO was found to enhance ZnO absorption properties as it shifts absorption region towards visible light [12]. CuO is a n-type semiconductor with relatively narrow band gap (1.2 eV), which is a key factor in enhancing the photocatalytic properties of coupled ZnO-CuO systems. However, the narrow band gap energy of CuO relates to rapid electron-hole recombination under light irradiation and, therefore, it requires stabilizing for improving its photocatalytic activity [13]. Furthermore, preparing ZnO-CuOx composites via oxidation of metallic substrates results in forming various morphologies of nanostructures that both components were proven to provide [14,15]. Nanostructuring the CuZn materials leads to significant development of their surface area, which is critical for catalysis. Moreover, during the process, numerous and diverse active sites are generated, such as grain boundaries and surface defects [16]. By that, surface rearrangement during oxidization improves adsorption properties and may impact both activity and selectivity of catalytic process.

The most common approach for synthesizing ZnO-CuO_x_ composites is still the hydrothermal method [17,18]. However, despite its feasibility, hydrothermal synthesis is a very time-consuming procedure for preparing this type of material [19]. Thus, other more sophisticated approaches for oxidized CuZn bimetallic systems formation have been developed and explored, such as plasma-assisted oxidation [20] or laser ablation [21]. One competitive method for CuZn oxidation that enables formation of nanostructured ZnO-CuOx films in a quick and facile way is electro-oxidation, i.e., anodizing. Applying an electrochemical approach allows for rapid generating of continuous oxide films on the electrode surface and ensures high control over the morphological features of formed layers. Raschi et al. [22] employed a brass anodization method for preparation of CuO-ZnO. They electrooxidized brass foil in alkaline media at 12 V, covering the electrode surface with a petal-like nanostructure composed of both semiconducting oxides. As was concluded, this specific morphology originates from the presence of crystalline ZnO which introduces polygonal forms to the mixed-oxide layer. Generally, brass anodization has not been yet widely researched and the scientific literature lacks reports that provide insight into anodic oxide growth mechanisms in such systems, especially at mildly oxidative conditions. There are few studies [23,24] that probe the subject; however, further understanding on how the morphological characteristics of formed passive layers relate to applied conditions is required. This might be of a considerable interest since CuZn-oxidized materials (such as CuO-ZnO mixed n-p semiconducting composite) draw significant attention and constantly find new applications in more advanced photochemical systems.

In light of the above, the present study was designed to explore in detail the electrochemical behavior of α-brass subjected to an electro-oxidation in alkaline electrolyte at mildly oxidative potentials. It investigates phase and morphology transitions along altering the potentials applied. Gaining a better understanding of the oxide layer formation process is crucial in improving and optimizing synthesis procedures for CuZn-derived photo- and electrocatalysts.

## 2. Materials and Methods

### 2.1. Material Preparation

The brass (Cu:63 Zn:37) sheet used in this study was purchased from Avanti (Tarnów, Poland) and cut into smaller 2 cm × 2 cm pieces. Brass samples were degreased and washed with acetone and ethanol, respectively. Brass electro-oxidation was carried out in glass single-compartment cell (Atlas Sollich, NSD50/23/80, Rębiechowo, Poland) connected to a potentiostat (Atlas Sollich 0531), where brass foil was mounted horizontally on the bottom of the cell. Area of the material, which was exposed to electrolytes, was limited to 1.77 cm^2^ by rubber O-ring seal. Brass passivation was performed in a three-electrode configuration where brass foil was used as a working electrode, Pt as a counter electrode, and Ag│AgCl│KCl_sat_ as a reference electrode. Here, all potential values are given versus Ag│AgCl│KCl_sat._ Electrolyte used in this study was 1 M NaOH solution. Brass passivation was tested at constant potentials from passive range for 10 min each. In order to investigate the potential influence on morphology of obtained oxide films, eight potential values from −0.2 V to 0.5 V range with 0.1 V increments were selected for this study. Passive range was established from cyclic voltammetry by plotting a polarization curve in typical Tafel’s coordinates (E vs. Log|j|), where E stands for applied potential and j stands for current density.

### 2.2. Materials Characterization

Field emission scanning electron microscopy (FE-SEM, Quanta 3D FE-SEM, FEI) was employed to examine morphology of the prepared materials.

X-ray diffraction (XRD) phase analysis of the fabricated materials was made with Rigaku ULTIMA IV diffractometer equipped with Co Kα radiation source within the 2θ range of 20°–90°, step size of 0.02° and an acquisition rate of 1° per minute. The acquired data were processed using Match! software, and the crystallographic phases were identified using COD crystallographic database.

X-ray photoelectron (XPS) analysis was carried out using a PHI-TFA XPS spectrometer (Physical Electronic Inc.), equipped with an X-ray Al-monochromatic source. The vacuum during XPS analysis was 10^−9^ mbar. The analyzed area was 0.4 mm in diameter and the analysis depth was 3–5 nm. Narrow multiplex scans of the peaks were recorded using a pass energy of 23.5 eV with a step size 0.1 eV, at a take-off angle of 45° with respect to the sample surface. Low energy electron gun was used for surface charge neutralization XPS. Spectra were processed using Multipak v8.0 (Physical Electronics Inc., Chanhassen, MN, USA). The elemental composition was determined from the XPS survey spectra. High-energy resolution spectra of O 1s and Zn 2p and Cu 2p photoelectron peaks were curve-fitted.

### 2.3. Photocatalysis Tests

Photocatalytic activity of the α-brass samples electro-oxidized at 0.1 V was determined by quantifying photodecomposition of methyl orange (MO) at room temperature (kept constant by a water recirculation system). The samples were immersed into 10 mL of 8 mg L^−1^ aqueous MO solution and placed on a perforated holder 5 mm above the bottom of the reactor and 25 cm under the source of irradiation. The solution was constantly stirred (300 rpm) throughout the photocatalysis measurements, with the magnetic stirrer placed underneath the sample holder. Prior to irradiation, the catalyst and solution were stirred in the dark for 30 min until adsorption–desorption equilibrium was achieved. Irradiation of the samples, lasting for 6 h, was conducted by using a Osram Vitalux lamp (300 W) that simulates solar spectrum and Philips HPR (125 W) Mercury lamp that provides UV light. After every hour, a fixed amount (1 mL) of the MO solution was removed for the absorption measurement at 464 nm (the maximum of the absorption spectra) using Agilent Carry 60 UV–Vis spectrophotometer, and after that returned to the solution. The absorbance was converted to MO concentration in accordance with the standard curve, showing a linear relationship between the concentration and the absorbance at this wavelength. Correspondingly, the MO adsorption in darkness with the presence of photocatalyst was also recorded every hour during 6 h. Prior to the measurement, MO solution was tested for photocatalysis in the absence of the photocatalyst to confirm its stability. The absence of change in MO concentration after 6 h of irradiation indicated its stability under the applied conditions and its degradation resulted only from the presence of a photocatalyst. The photocatalytic activity was tested and averaged for 5 samples for each irradiation source.

## 3. Results and Discussion

### 3.1. Materials Characterization

**Figure 1:**
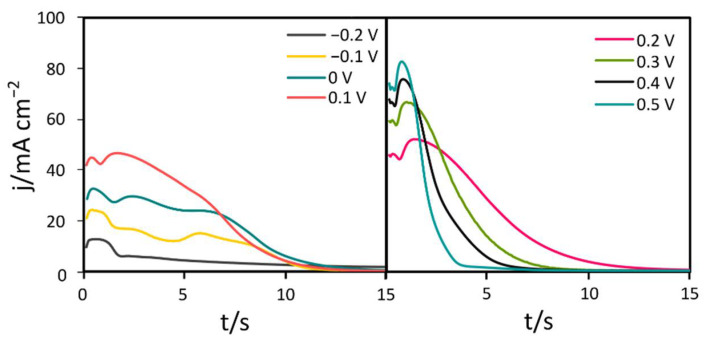
Initial phase of chronoamperometric j-*t* curves recorded for CuZn passivation in 1 M NaOH.

To investigate the electrochemical behavior of a CuZn surface subjected to passivation in highly alkaline media, current density vs. time (j-*t*) relation curves of each tested potential value were recorded with high resolution. As presented in Figure 1, the shape of j-*t* curves represents chemical transformations the CuZn surface undergoes during the initial phase of the process. The first decline of current density after electrode polarization can be observed within the first 2 s of the process irrespective of potential applied. This initial current drop represents covering the exposed metal surface with the first atomic layer of oxide, patching surface spots that were not covered with native oxide. The width of the peak representing this initial current decline narrowed down as more oxidative conditions were used. This indicates more rapid formation of the first atomic layer with more oxidative potential. In the next step, current density increases, implying breaking the previously formed native oxide layer. After the j-*t* curve reaches its peak value once more, current density starts to decline again which results from passive layer formation and its further propagation. The time in which this stage of the process occurs is also dependent on the potential applied. The more noble the value of potential, the more rapid the passive layer formation, that in turn prohibits current flow through the system. When the potential applied was lower than 0.3 V, current density dropped to minimal values within 10 to 15 s. Processes conducted under higher constant potential led to formation of a complete passive layer within 5 to 10 s.

**Figure 2:**
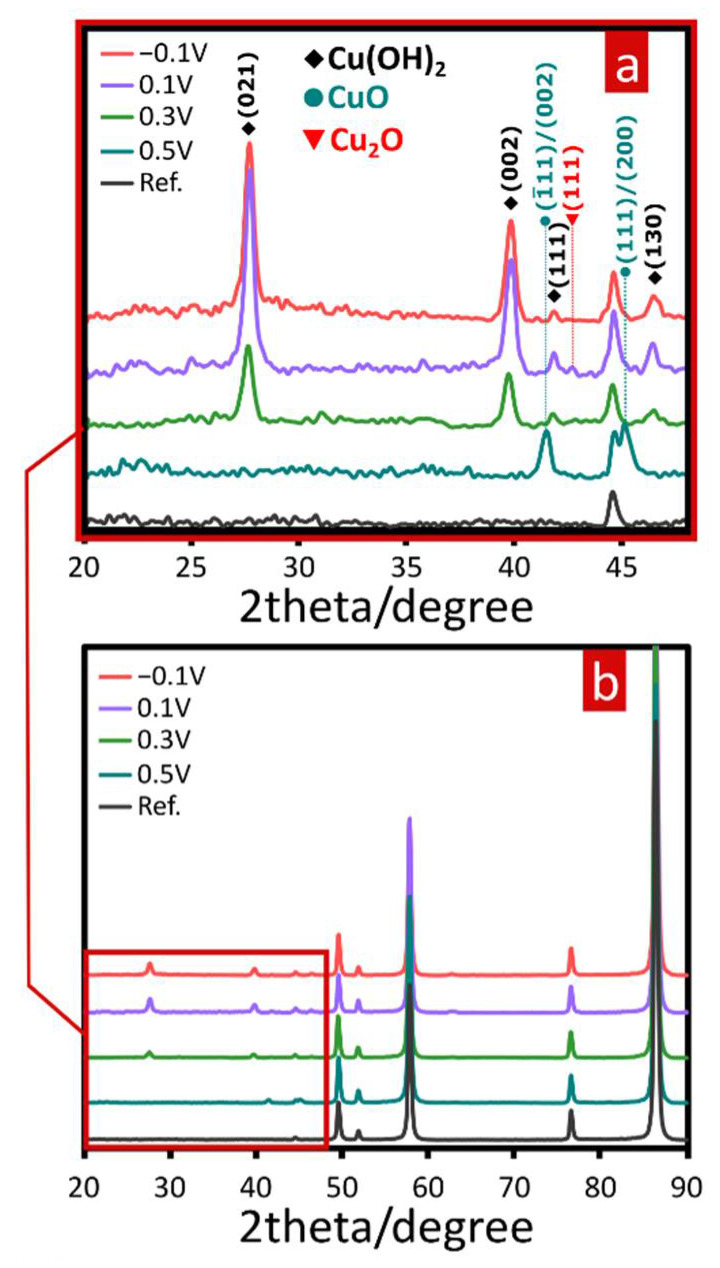
XRD patterns of as-prepared CuZn-based materials by potentiostatic oxidation at different applied potential values (V vs. Ag│AgCl│KCl_sat_) within (**a**) narrow and (**b**) wide 2 theta angle range.

Figure 2 shows diffractograms of α-brass samples subjected to potentiostatic oxidation in 1 M NaOH under selected applied potentials. To facilitate phase analysis of prepared materials and enable clear comparison, a reference sample of the brass substrate was also included in the analysis. As shown in Figure 2b, the diffraction pattern for the substrate represented as a black bottom line, indicating peaks for α-brass, can be found in each oxidized sample. Appearance of additional peaks suggesting formation of new crystalline phases can be spotted within the narrower 2 theta angle range (Figure 2a), therefore in a typical range for Cu and Zn oxidation products. As was found, electro-oxidative treatment of CuZn under reported conditions led mainly to formation of Cu(II)-based crystalline phases. Furthermore, there is a visible influence of applied potential on the phase composition of synthesized materials. When lower values of potential were applied, Cu(OH)_2_ (spertiniite) was found to be a dominant crystalline component of grown film. Two relatively intensive peaks at 27.7° and 39.8°, as well as two more minor peaks at 41.8° and 46.2°, match very well to reflections from (021), (002), (111) and (130) planes of Cu(OH)_2_, respectively. This pattern appeared in samples prepared under lower onset potentials (from –0.1 V to 0.3 V). Within this range of applied potential, some traces of cuprite (Cu_2_O) can also be detected since minor peaks at 43.2° assigned to reflection from (111) cuprite plane might be included as relevant findings of the analysis. In the case of samples prepared at higher applied potential (0.5 V), crystalline composition differs from other synthesized materials. There are only two peaks at 41.5° and 45.1° that match reflections from CuO (tenorite) planes: (111) or (002) and (−111) or (200), respectively. These results suggest valid implications concerning the electro-oxidation sequence of CuZn alloy in highly alkaline media. As XRD analysis proved, Cu(OH)_2_ is a dominant phase with a small amount of Cu_2_O in samples prepared when the constantly applied potential was equal or more negative than 0.3 V. Above this value, in samples prepared under more oxidative conditions, solely CuO is present in the oxide layer. These findings represent consecutive oxidation reactions that the CuZn surface was involved in within the presented system.

**Figure 3:**
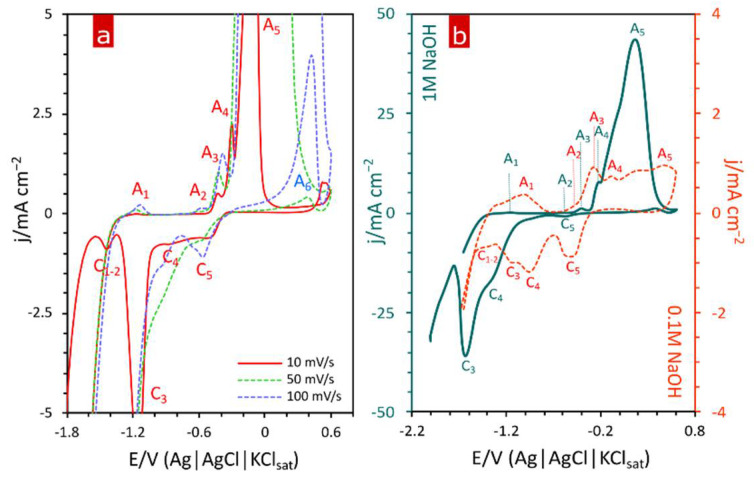
Cyclic voltammetry investigation of α-brass in alkaline media: (**a**) voltammograms recorded at various sweep rates showing anodic (A) and cathodic (C) peaks in 1 M NaOH and (**b**) comparison of voltammograms for the alloy in different concentrations of NaOH (dark blue line—1 M; red dashed line—0.1 M) at 70 mV s^−1^.

Thorough electrochemical analysis using cyclic voltammetry (CV) revealed the oxidation sequence of CuZn alloy components when subjected to electro-oxidation in 1 M NaOH solution. The first anodic peak (A_1_) that can be noticed in presented voltammograms (Figure 3) appears at approx. −1.16 V and originates from dissolution activation of less noble component-zinc and its subsequent bonding with hydroxide ions [25,26]:

Zn_ads_ + OH^−^ → Zn_ads_OH + e^−^, (1)

Zn_ads_OH + OH^−^ → ZnO + H_2_O + e^−^ (2)

As the potential increases, the initially formed first type of oxidized zinc undergoes further oxidation leading to formation of ZnO as represented by Equation (2). This step of brass oxidation can be observed as anodic peak A_2_ in recorded cyclic voltammograms (Figure 3). The potential region limited by those two Zn oxidation peaks can be referred to as preferential dissolution of the less noble component in alloy systems. As has been proven [27], preferential dissolution during brass electro-oxidation causes interdiffusion rearrangement within the alloy as Zn migrates from bulk to surface of the material. Transformation of type I oxidized zinc form to type II is accompanied by color change of brass surface. Dehydration in highly alkaline media results in increased zinc concentration within oxide layer and its grey color intensifies towards black as Zn concentration increases [28]. While the potential continues to increase towards more noble values, a characteristic critical potential (E_crit_) is reached.

At this point, preferential dissolution ends and both components of the alloy undergo oxidation. It can be observed as significant current increase as both Zn and Cu dissolve and go through further transformations into consecutive forms. Therefore, E_crit_ is determined by a beginning of the first Cu oxidation peak marked as A_3_ in Figure 3. This peak is related to the first step of Cu oxidation, namely formation of a thin porous layer of Cu_2_O on the metal surface as represented by Equation (3). As grown pores expand in time, part of Cu_2_O is dissolved (Equation (4)). This process, together with metal dissolution as Cu^2+^ (Equation (5)) occurring inside the oxide pores, leads to initialization of Cu(OH)_2_ formation. Therefore, a subsequent anodic peak A_4_ is assigned to nucleation and growth of Cu(OH)_2_ phase.

2Cu + 2OH^−^ → Cu_2_O + H_2_O + 2e^−^ (3)

Cu + nOH^−^ → Cu(OH)^2−n^_n_ + 2e^−^ (4)

Cu_2_O + H_2_O + (2n−2)OH^−^ → 2Cu(OH)^2−n^_n_ + 2e^−^ (5)

The final stage of Cu oxidation relies on transformation of both Cu_2_O and Cu(OH)_2_ into CuO. This phase is represented as peak A_5_ in Figure 3. CuO seems to be a stable phase since the current decreased with potential shifting to more noble values. It can be concluded that CuO formed a passive layer on the electrode surface. This analysis was supported by XRD findings showing dependence of applied potential on phase composition of oxide layers grown during potentiostatic oxidation of α–brass conducted in this study. Obtained diffractograms indicated consecutive transformation of Cu_2_O into Cu(OH)_2_ and finally into CuO with increasing applied potential.

**Figure 4:**
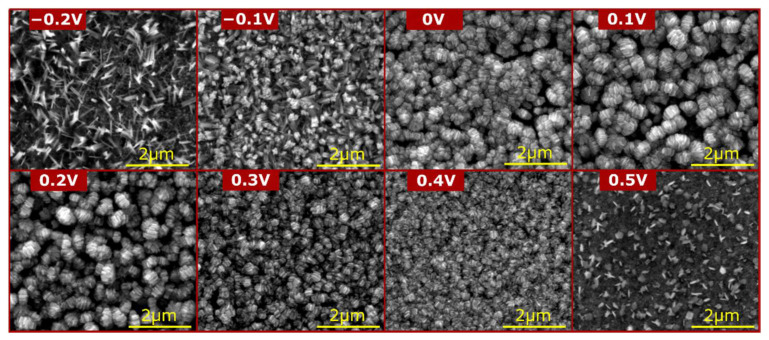
SEM images displaying morphology obtained during potentiostatic oxidation of CuZn alloy in alkaline media at various applied potentials.

**Figure 5:**
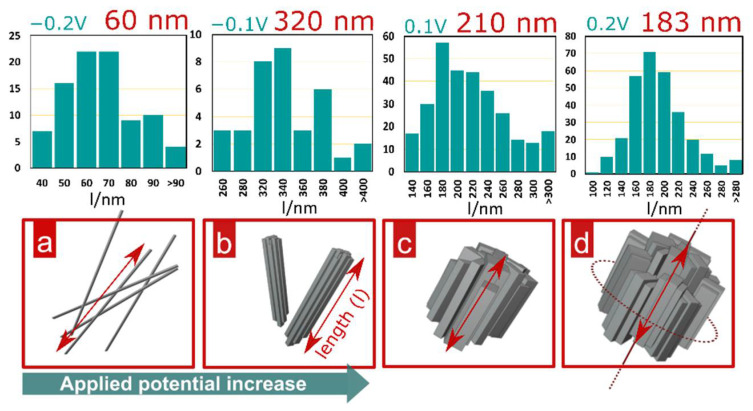
Histograms representing length distribution of prepared barrel-like structures under given applied potentials and conceptual representation of consecutive phases of CuZn barrels formation: (**a**) nanowires; (**b**) nanowires agglomerates; (**c**) barrel-like particles and (**d**) fully grown barrel-like particles.

As presented in Figure 4, the morphology of the CuZn alloy surface after electro-oxidation in 1 M NaOH depends on the applied potential. When the brass sample was subjected to passivation under the least noble potential (−0.2 V) from the tested range, the process yielded nanoneedles morphology. This type of nanostructure is characteristic for copper electro-oxidation in alkaline media [29]. During the process, crystallization nuclei are formed on the metal surface exposed to electro-oxidation in alkaline media defining spots for nanostructure growth and expansion. As the surface continues to be oxidized, crystallization progresses perpendicularly from the surface plane. At some point, after reaching considerable height, formed Cu(OH)_2_ nanoneedles fall down, leaving morphology that can be observed in Figure 4 for the sample oxidized at −0.2 V and schematically represented by Figure 5a. Further experiments conducted at more noble constant potentials revealed the evolution of the formed nanostructure. When applying potential of −0.1 V and higher, Cu(OH)_2_ nanoneedle formation is intensified, which results in more densely packed morphology of thicker nanowires. Increased density of superficially grown crystalline structures with wider cross-sectional diameters led to formation of nanowires agglomerates as implied by SEM observations of the sample electrooxidized at −0.1 V shown in Figure 4.

As suggested by SEM micrographs for samples prepared at higher potentials (0–0.2 V), the aspect ratio of grown nanowires increases as applied potential increases. More rapid growth of thicker Cu(OH)_2_ nanowires causes its faster detachment from the surface. Thicker and shorter nanocrystals undergo further crystallization, expanding their sectional diameter (Figure 5c,d). This leads to formation of barrel-like agglomerates that originate from initially grown cupric hydroxide nanostructures. The size of barrel-like agglomerates was found to strongly depend on applied potential. Constant expanding of their diameters along with the potential increase was disrupted with the sample electrooxidized at 0.3 V. Above this value, the size of barrel agglomerates decreased when passivation was carried out at more noble potentials. Such an outcome may be explained by the fact that more oxidative conditions accelerate surface architecture rearranging (Figure 1), and thus nanoneedle growth, their subsequent agglomeration and eventual detachment occurs more rapidly. Therefore, faster rearranging leads to formation of smaller barrel-like agglomerates but in significantly higher quantity. Drastic alteration of the morphology was found in the sample passivated at the most oxidative applied potential tested in this study, i.e., at 0.5 V. In this case, it is clear that the oxide structure followed a different growth mechanism as the surface exhibited initial phase of a flower-petal-like morphology formation (Figure 4, sample prepared at 0.5 V). Such morphology is characterized by polygonal flat nanocrystals resembling rose petals. This finding suggests the contribution of a ZnO crystalline phase in forming the passive layer on the CuZn surface [30]. ZnO crystallizes in a hexagonal wurtzite-type structure [31], which might be recognized as an origin for polygonal morphology obtained during CuZn electro-oxidation at 0.5 V. Taking this justification into account, it can be stated that 0.5 V is a sufficiently high potential value required to trigger ZnO crystallization in the presented system. Furthermore, it implies that at less noble potentials Zn oxidation yields an amorphous phase that cumulates on the brass surface as a compact oxide layer.

**Figure 6:**
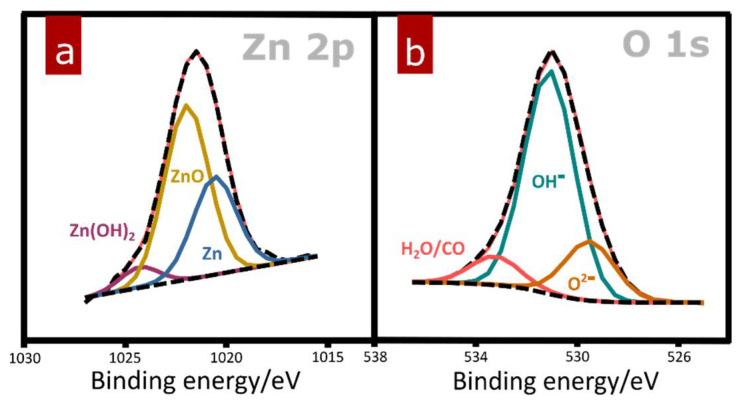
High-resolution XPS spectra for (**a**) Zn 2p and (**b**) O 1s regions recorded for a sample passivated at 0.1 V.

To confirm the presence of amorphous ZnO phase in samples passivated at lower potentials in the tested range, a CuZn sample electro-oxidized at 0.1 V was analyzed using X-ray photoelectron spectroscopy. High-resolution spectra for Zn 2p region and O 1s region are presented in Figure 6. Analyses of fitted spectra revealed the presence of zinc oxide in the tested sample (Figure 6a). Considering that XRD analysis did not show any traces of crystalline ZnO and SEM observation did not provide any structural suggestions on wurtzite formation at potentials below 0.5 V, XPS findings clearly indicate that an amorphous form of a less noble Zn component in the system is generated. As shown in Figure 6b, oxygen was found to contribute mainly as hydroxide. However, part of the oxygen species was identified as oxide. This outcome is in line with the conclusion that the passive film created under studied conditions is composed of two components: crystalline Cu(OH)_2_ and amorphous ZnO, as suggested by XRD and XPS techniques, respectively. Therefore, brass electro-oxidation within the passive range (from −0.2 V to 0.5 V) in highly alkaline media leads to formation of a semi-crystalline hybrid oxide film. As proven by various reports (Table 1), to achieve complete copper oxidation and form crystalline ZnO, brass electro-oxidation has to be performed in more aggressive conditions.

### 3.2. Photocatalytic Activity

**Figure 7:**
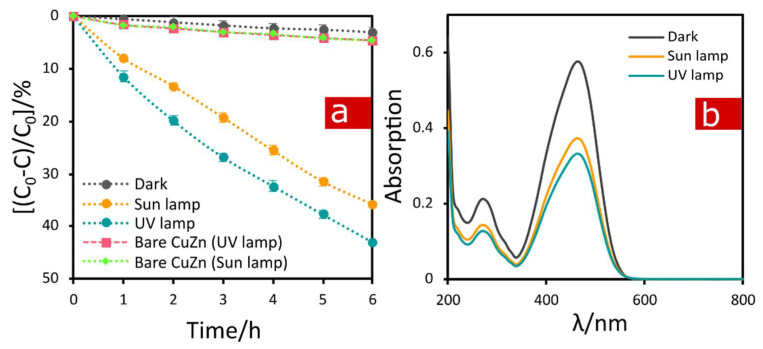
Photoactivity of CuZn electrooxidized at 0.1 V compared to bare CuZn in methyl orange (MO) degradation under two different sources of irradiation: UV and sunlight lamps; (**a**) evolution of quantitative changes of MO conversion in time and (**b**) UV-Vis adsorption spectra for MO after 6 h of irradiation in a presence of CuZn-based catalyst.

Figure 7 shows the absorption and degradation behavior of MO with the electrooxidized α-brass samples as catalyst. The results are given as ((C_0_-C)/C_0_), where C_0_ was the initial MO concentration and C was the MO concentration after a given time in the dark, and under two different irradiation sources. The samples show decent photocatalytic activity, which increases with time. However, the usage of the UV lamp increases the photocatalytic activity from the value of 36% to the value of 43%, both at 6 h of irradiation. Photocatalytic activity of the bare α-brass samples was also tested and is presented in Figure 7a for reference. The absorption was quite small compared to the photoactivity, i.e., the increase in MO photodegradation is the result of the photocatalytic reactions in the presence of the formed nanostructures and the change in surface morphology, an increase in surface area accessible to the photocatalysis and number of photocatalitically active sites that facilitate dye molecules and reactive oxygen species (ROS) diffusion and transportation [35].

**Figure 8:**
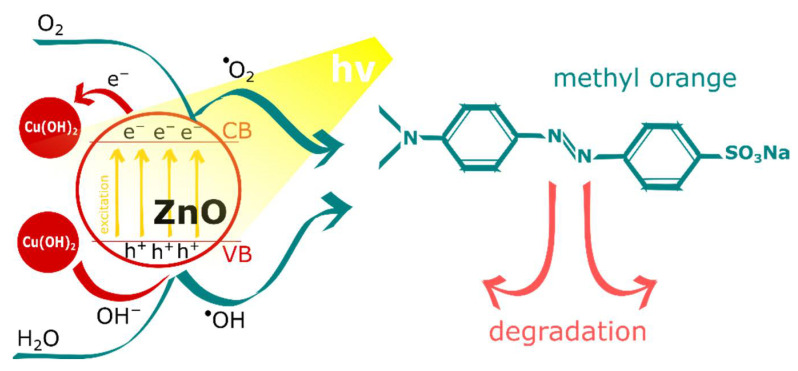
Schematic representation of MO photodegradation mechanism in a presence of Cu(OH)_2_-ZnO hybrid material.

The mechanism of MO photodegradation with Cu(OH)_2_-ZnO material can be understood from Figure 8. Zinc oxide as an n-type semiconductor with a band gap of 3.37 eV was found to be active in photoinduced degradation of organic compounds [36]. Because of its wide band gap, ZnO exhibits photoactivity practically only under UV light irradiation. Commonly used methods for enhancing the photocatalytic efficiency of ZnO are based on extending its optical absorption to the visible region as well as inhibition of the recombination of photogenerated electron-hole pairs [37]. Photoactivity of ZnO originates from electron-hole separation that occurs due to light excitation. By that, excited electrons (e^−^) are transferred to and occupy conducting band (CB), whereas holes (h^+^) are formed on valance band (VB) [38]. Both of these species are involved in the photodegradation process as they are responsible for generation of radicals causing degradation of organic compounds. Thus, under light irradiation, excited electrons and formed holes trigger generation of superoxide anion radicals (^●^O_2_^−^) and hydroxyl radicals (^●^OH), respectively. Those consecutive steps of photoinduced MO degradation by ZnO can be represented by Equations (6)–(9):

ZnO + hν → ZnO (h^+^ + e^−^) (6)

O_2_ + e^−^ → ^●^O_2_^−^ (7)

H_2_O + h^+^ → ^●^OH + H^+^ (8)

h^+^ + ^●^O_2_^−^ + ^●^OH + MO → CO_2_ + H_2_O (9)

The presence of Cu(OH)_2_ should also be recognized as beneficial for ZnO photoactivity in the process. On the one hand, neighboring cupric hydroxide structures may act as electron acceptors stabilizing photoinduced electron-hole separation [39,40]. As Yu and Ran [41] confirmed, by using fluorescence quenching experiments, photogenerated electrons can be transferred from TiO_2_ to Cu(OH)_2_ formed in a close surrounding to photoactive semiconducting oxide. Thus, cupric hydroxide traps photoexcited electrons sustaining and facilitating radical generation on valance band. Simultaneously, by accepting electrons, Cu(OH)_2_ undergoes partial reduction to metallic copper, yielding mixed Cu^2+^/Cu^0^ clusters. On the other hand, the surface of the Cu(OH)_2_ structure may adsorb –OH and, therefore, act as an heterogenous catalyst providing and consequently accelerating formation of hydroxyl radicals as main agents triggering MO decomposition [42]. Moreover, this effect can be intensified, as cupric hydroxide reduction initiated by photoelectrons releases OH^−^ ions that can be included as an additional source for radical formation.

## 4. Conclusions

Electrochemical passivation of commercially available α–brass in highly alkaline media can be used as an inexpensive and facile method for preparing a nanostructured material active in photodegradation of an organic dye (methyl orange). Thorough analysis of electrochemical behavior and phase composition provided interesting findings on the oxidation sequence that brass surface follows when subjected to passivation in 1 M NaOH. It was found that potentiostatic oxidation at mild conditions results in formation of a thin semi-crystalline Cu(OH)_2_-ZnO layer on brass surface at potentials lower than 0.5 V vs. Ag│AgCl│KCl_sat_. The grown oxide films indicated novel morphology of barrel-like structures observed for the first time in the brass surface oxidation. Additionally, the size of these structures can be adjusted by changing the applied potential value varying surface area of the material, which can find application in preparing brass-based electrodes for electrocatalytic purposes. By tuning the applied potential, the presented method can be used for formation of a nanostructured material of three different morphologies on α-brass surface. Nanowire morphology consisting mainly of Cu(OH)_2_ can be grown under the least oxidative range, namely ≤−0.2 V. Above this value (from −0.1 V to 0.4 V), the system indicates morphology of barrel-like structures of crystalline Cu(OH)_2_ and amorphous ZnO. At potentials exceeding 0.5 V, polygonal petal-resembling crystals are formed, implying crystallization of ZnO. A hybrid Cu(OH)_2_-ZnO electrode was used in methyl orange (MO) photodegradation, yielding moderate activity under both UV and simulated daylight irradiation. Widening ZnO absorption towards visible light might be caused by the presence of Cu(OH)_2_. Cupric hydroxide can act as a trap for photoexcited electrons inhibiting electro-hole pairs recombination.

## Figures and Tables

**Table 1:** Literature reports on CuZn electro-oxidation with process conditions and yielded morphologies.

Substrate	Film Composition after Electro-Oxidation	Morphology	Electrolyte Composition	Voltage	Ref
CuZn	CuO-ZnO (after annealing)	particles	1 M oxalic acid	40 V (25 min)	[32]
CuZn (65:35)	CuO-ZnO	flowers, petals cubes	0.05, 0.1, 0.2 M NaOH and KOH (with NH_4_Cl, NH_4_F, NH_4_SO_4_)	6, 12, 24 V (15, 30, 60 min)	[30]
CuZn (70:30)	CuO-ZnO; Cu-Zn-O	flowers	0.1 M, 1 M NaHCO_3_	12 V (5 min)	[33]
CuZn	CuO-ZnO	spherical structure	1 M NaOH	30–60 V (10 min)	[34]
CuZn (65:35)	CuO-ZnO	flowers	0.1 M NaOH + 0.025 M NH_4_Cl	12 V (15–45 min)	[22]
CuZn (63:37)	Cu(OH)_2_-ZnO	barrels	1 M NaOH	(−0.2)–0.5 V (10 min)	This study

## Data Availability

Not applicable.
